# Low-Density Lipoprotein Cholesterol Levels and Bleeding Risk in Venous Thromboembolism

**DOI:** 10.1001/jamanetworkopen.2025.9467

**Published:** 2025-05-09

**Authors:** Carmine Siniscalchi, Tiziana Meschi, Pierpaolo Di Micco, Egidio Imbalzano, Luis Hernández-Blasco, Isabelle Mahé, José Luis Fernández-Reyes, Alberto García-Ortega, Peter Verhamme, Joaquín Alfonso-Megido, Manuel Monreal, and the RIETE Investigators

**Affiliations:** 1 Department of Internal Medicine, Parma University Hospital, Parma, Italy; 2 AFO Medicina PO Santa Maria delle Grazie, Pozzuoli Naples Hospital 2 Nord, Naples, Italy; 3 Division of Internal Medicine, Department of Clinical and Experimental Medicine, University of Messina, Messina, Italy; 4 Pneumology Department of Clinical Medicine, Miguel Hernández University, ISABIAL, Dr. Balmis University General Hospital, Alicante, Spain; 5 Department of Internal Medicine, Hôpital Louis Mourier, Assistance Publique des Hôpitaux de Paris (AP-HP), Colombes, France; 6 Université Paris Cité, France. INSERM UMR-S-1140, Paris, France.; 7 Department of Internal Medicine, Complejo Hospitalario de Jaén, Jaén, Spain; 8 Department of Pneumonology, Hospital Universitario Doctor Peset, Valencia, Spain; 9 Vascular Medicine and Haemostasis, University of Leuven, Leuven, Belgium; 10 Hospital Valle del Nalón, Department of Internal Medicine, Langreo, Asturias, Spain; 11 Chair for the Study of Thromboembolic Disease, Faculty of Health Sciences, UCAM - Universidad Católica San Antonio de Murcia, Murcia, Spain; 12 CIBER Enfermedades Respiratorias (CIBERES), Madrid, Spain

## Abstract

This case-control study evaluates low-density lipoprotein cholesterol levels and bleeding risk during anticoagulation in patients with acute venous thromboembolism.

## Introduction

Low-density lipoprotein cholesterol (LDL-C) levels are a well-established therapeutic target for cardiovascular risk reduction, but their role in hemostasis remains less understood.^1,2^ While previous studies^3,4,5^ suggest that aggressive LDL-C lowering may increase bleeding risk in patients with arterial disease, this association has not been explored in patients receiving anticoagulation for venous thromboembolism (VTE). We sought to evaluate LDL-C levels and bleeding risk during anticoagulation in patients with acute VTE.

## Methods

We conducted a case-control analysis using data from the RIETE (Registro Informatizado Enfermedad TromboEmbólica) registry, a large multicenter, observational registry enrolling consecutive patients with objectively diagnosed VTE.^6^ The study was conducted in accordance with the declaration of Helsinki. Ethics committee approval was obtained from Comité de Ética de la Investigación del Hospital Universitario Germans Trias i Pujol (Badalona, Spain). All participants provided written informed consent. The study included patients from March 2009 to July 2024 who had available baseline LDL-C levels. Because measuring LDL-C is not routine, 75.6% of patients did not have available LDL-C values. Therefore, we checked for potential selection bias by comparing baseline characteristics of patients with and without LDL-C measurements and found no major differences among patients without LDL-C measurements (eTable in Supplement 1). Bleeding events during the first 90 days of anticoagulation were classified as major or nonmajor bleeding according to standard definitions. We reported standardized differences between patients in both subgroups. Standardized differences greater than 0.1 in absolute value were considered relevant. Multivariable Cox proportional hazards regression models, adjusted for competing risks using the Fine-Gray method, were used to assess LDL-C levels and bleeding outcomes. This study followed the STROBE reporting guideline. Data were analyzed using SPSS version 20 (IBM Corp). To identify comparisons of clinical relevance, we reported standardized differences between patients in both subgroups. A standardized difference greater than 0.1 in absolute value was considered relevant.

## Results

Among 19 237 patients with available LDL-C levels, 2502 (13.0%) had LDL-C levels less than 70 mg/dL (to convert to mmol/L, multiply by 0.0259). Compared with those with LDL-C levels of 70 mg/dL or higher, these patients were older, more frequently male, and had a higher prevalence of hypertension, diabetes, prior arterial disease, anemia, and active cancer (Table 1). Compared with those with LDL-C levels of 70 mg/dL or higher, these patients were older (standardized difference, 0.228), more frequently male (standardized difference, 0.125), and had a higher prevalence of hypertension (standardized difference, 0.254), diabetes (standardized difference, 0.355), prior arterial disease (standardized difference, 0.144), anemia (standardized difference, 0.436), and active cancer (standardized difference, 0.159). During the first 90 days of anticoagulation, 743 patients (3.9%) experienced bleeding events: 294 major bleeding, 449 nonmajor bleeding, and 32 fatal bleeding cases (Table 2). Patients with LDL-C levels less than 70 mg/dL had an increased risk of overall bleeding (adjusted hazard ratio [AHR], 1.40; 95% CI, 1.16-1.69) and nonmajor bleeding (AHR, 1.49; 95% CI, 1.17-1.90), with hematomas being the most frequent bleeding site (AHR, 2.11; 95% CI, 1.49-2.98). The increased bleeding risk was observed early during anticoagulation and was independent of statin use.

**Table 1.** Baseline Characteristics of Patients According to LDL-C Levels

Characteristic	LDL-C level, No. (%)		Standardized difference
	<70 mg/dL (n = 2502)	≥70 mg/dL (n = 16 735)	
Demographic			
Sex			
Female	1088 (43)	8322 (49.7)	0.125
Male	1414 (57)	8413 (50.3)	0.125
Age, mean (SD), y	69 (17)	65 (17)	0.228
BMI, mean (SD)	28 (6.0)	29 (5.7)	0.136
Outpatients	1540 (64)	11 388 (70)	0.137
Initial VTE presentation			
Pulmonary embolism	1694 (68)	10 179 (61)	0.144
Lower-limb DVT	661 (26)	5836 (35)	0.184
Upper-limb DVT	147 (5.9)	720 (4.3)	0.072
Comorbidities			
Chronic lung disease	332 (13)	1698 (10)	0.097
Hypertension	1516 (61)	8045 (48)	0.254
Diabetes	720 (29)	2409 (14)	0.355
Prior myocardial infarction	373 (15)	989 (5.9)	0.299
Prior ischemic stroke	263 (11)	965 (5.8)	0.174
Peripheral artery disease	159 (6.4)	550 (3.3)	0.144
Recent major bleeding	98 (3.9)	322 (1.9)	0.119
Serum lipid levels			
Total cholesterol	125 (27)	189 (39)	1.886
LDL-cholesterol	58 (8.1)	118 (32)	2.540
HDL-cholesterol	44 (22)	46 (15)	0.090
Triglycerides	128 (73)	137 (65)	0.134
Risk factors for bleeding			
Active cancer	399 (16)	1767 (11)	0.159
Liver cirrhosis	27 (1.1)	49 (0.3)	0.095
Gastroduodenal ulcer	44 (1.8)	163 (1.0)	0.068
Anemia	1146 (46)	4247 (25)	0.436
Leukocyte count >11 000/μL	801 (32)	4146 (25)	0.161
Platelet count <100 000/μL	83 (3.3)	286 (1.7)	0.103
Abnormal prothrombin time	369 (16)	1068 (7.0)	0.294
CrCl levels <60 mL/min	1052 (42)	4804 (29)	0.282
Prognostic scores for bleeding			
RIETE, high-risk (≥4 points)	439 (18)	1191 (7.6)	0.320
VTE-BLEED, high-risk (≥2 points)	1553 (63)	7306 (45)	0.371
Modified ACCP, high-risk (≥2 points)	1641 (71)	7856 (50)	0.436
DOAC, high-risk (≥8 points)	338 (19)	1080 (9.0)	0.287

Abbreviations: ACCP, American College of Chest Physicians; BMI, body mass index (calculated as weight in kilograms divided by height in meters squared); CrCl, creatinine clearance; DOAC, direct oral anticoagulant; DVT, deep vein thrombosis; HDL, high-density lipoprotein: LDL, low-density lipoprotein: RIETE, Registro Informatizado Enfermedad TromboEmbólica; VTE-BLEED, venous thromboembolism-bleedings.

SI conversion factors: To convert CrCl level to milliliters per second per meter squared, multiply by 0.0167; HDL and LDL from mg/dL to mmol/L, multiply by 0.0259; platelets to ×10^9^/L, multiply by 1; triglycerides from mg/dL to mmol/L, multiply by 0.0113; and WBCs to cells ×10^9^/L, multiply by 0.001.

**Table 2.** Rates of Events During the First 90 Days According to LDL-C Levels at Baseline

Event	LDL-C level, No. (%)		Odds ratio (95%CI)
	<70 mg/dL (n = 2502)	≥70 mg/dL (n = 16 735)	
Overall bleeding	158 (6.31)	585 (3.50)	1.86 (1.55-2.23)
Major bleeding	62 (2.48)	232 (1.39)	1.81 (1.36-2.40)
Gastrointestinal	16 (0.64)	75 (0.45)	1.43 (0.83-2.46)
Hematoma	23 (0.92)	53 (0.32)	2.92 (1.79-4.77)
Intracranial	11 (0.44)	39 (0.23)	1.89 (0.97-3.70)
Retroperitoneal	5 (0.20)	20 (0.12)	1.67 (0.63-4.46)
Urinary	2 (0.08)	12 (0.07)	1.11 (0.25-4.98)
Uterine	1 (0.04)	11 (0.07)	0.61 (0.08-4.71)
Other sites	4 (0.16)	22 (0.13)	1.22 (0.42-3.53)
Nonmajor bleeding	96 (3.84)	353 (2.11)	1.85 (1.47-2.33)
Hematoma	24 (0.96)	76 (0.45)	2.12 (1.34-3.37)
Urinary	18 (0.72)	91 (0.54)	1.33 (0.80-2.20)
Gastrointestinal	23 (0.92)	70 (0.42)	2.21 (1.38-3.54)
Uterine	4 (0.16)	20 (0.12)	1.34 (0.46-3.92)
Other sites	27 (1.08)	96 (0.57)	1.89 (1.23-2.90)
Fatal bleeding	11 (0.44)	21 (0.13)	3.51 (1.69-7.30)
Intracranial	4 (0.16)	10 (0.06)	2.68 (0.84-8.55)
Retroperitoneal	2 (0.08)	5 (0.03)	2.68 (0.52-13.8)
Gastrointestinal	2 (0.08)	3 (0.02)	4.46 (0.75-26.7)
Hematoma	1 (0.04)	0	NA
Other sites	2 (0.08)	3 (0.02)	4.46 (0.75-26.7)
Nonbleeding deaths	180 (7.2)	533 (3.2)	2.36 (1.98-2.81)
Pulmonary embolism	10 (0.40)	31 (0.19)	2.16 (1.06-4.42)
Disseminated cancer	48 (1.9)	190 (1.1)	1.70 (1.24-2.34)
Infection	22 (0.88)	48 (0.29)	3.08 (1.86-5.12)
Heart failure	17 (0.68)	30 (0.18)	3.81 (2.10-6.92)
Respiratory failure	11 (0.44)	49 (0.29)	1.50 (0.78-2.90)
Multiorganic failure	18 (0.72)	48 (0.29)	2.52 (1.46-4.34)
Bronchoaspiration	10 (0.40)	10 (0.06)	6.71 (2.79-16.1)
Unknown	16 (0.64)	42 (0.25)	2.56 (1.44-4.56)
Other	28 (1.1)	85 (0.51)	2.22 (1.44-3.41)

Abbreviations: LDL-C, low-density lipoprotein cholesterol; NA, not applicable.

SI conversion factors: To convert LDL from mg/dL to mmol/L, multiply by 0.0259.

## Discussion

Study limitations include the high proportion of missing LDL-C data due to the lack of routine lipid testing. However, the lack of important baseline differences between included and excluded patients is notable. Additionally, our multivariable analysis confirmed that the association between LDL-C levels and bleeding risk was independent of these variables. Another limitation is the inability to account for LDL-C fluctuations over time, which may have affected bleeding risk, particularly in patients undergoing lipid-lowering therapy adjustments.

In this study, low LDL-C levels were associated with an increased risk of bleeding, particularly hematomas, in patients receiving anticoagulation for VTE. Given that LDL-C is not currently considered in bleeding risk stratification, our findings suggest a potential new factor for risk assessment in this population. Future studies should explore whether LDL-C levels can refine existing bleeding risk models and whether strategies to mitigate this risk are warranted.

## Notes

Supplement 1. **eTable.** Baseline Characteristics in Patients With- Versus Without Information on LDL-C Levels

Supplement 2. **Nonauthor Collaborators.** The RIETE Investigators

Supplement 3.
Data Sharing Statement
