# Supplementary material for: Low-Density Lipoprotein Cholesterol Levels and Bleeding Risk in Venous Thromboembolism
Source: JAMA Netw Open. 2025 May 9;8(5):e259467. doi: 10.1001/jamanetworkopen.2025.9467 (PMC12065040; doi:10.1001/jamanetworkopen.2025.9467)
Supplement: Supplement 1. — eTable. Baseline Characteristics in Patients With- Versus Without Information on LDL-C Levels [file jamanetwopen-e259467-s001.pdf]

Supplemental Online Content

Siniscalchi C, Meschi T, Di Micco P, et al; for the RIETE Investigators. Low-density lipoprotein cholesterol levels and bleeding risk in venous thromboembolism. *JAMA Netw Open.* 2025;8(5):e259467. doi:10.1001/jamanetworkopen.2025.9467

eTable. Baseline Characteristics in Patients With- Versus Without Information on LDL-C Levels

This supplemental material has been provided by the authors to give readers additional information about their work.

eTable. Baseline Characteristics in Patients With- Versus Without Information on LDL-C Levels

	LDL-c levels	No LDL-c levels	Standardized differences
Patients, N	***19,237***	***59,759***	
**Demographics,**			
Female	9,410 (49%)	30,201 (51%)	0.032
Age, mean years±SD	65±17	65±18	0.038
BMI, mean Kg/m^2±SD	29±5.7	28±5.9	0.102
Outpatient	12,928 (69%)	40,553 (71%)	0.031
**Initial VTE presentation,**			
Pulmonary embolism	11,873 (62%)	34,147 (57%)	0.093
Lower-limb DVT	6,497 (34%)	22,610 (38%)	0.085
Upper-limb DVT	867 (4.5%)	3,002 (5.0%)	0.024
**Comorbidities,**			
Chronic lung disease	2,030 (11%)	6,851 (11%)	0.029
Hypertension	9,561 (50%)	27,633 (46%)	0.067
Diabetes	3,129 (16%)	9,278 (16%)	0.019
Prior myocardial infarction	1,362 (7.1%)	3,772 (6.4%)	0.030
Prior ischemic stroke	1,228 (6.4%)	3,680 (6.2%)	0.008
Peripheral artery disease	709 (3.7%)	1,935 (3.3%)	0.023
Recent major bleeding	420 (2.2%)	1,485 (2.5%)	0.020
**Serum lipid levels,**			
Total cholesterol	181±43	175±43	0.142
LDL-cholesterol	111±36	-	-
HDL-cholesterol	46±16	46±49	0.008
Triglycerides	136±66	140±89	0.050
**Risk factors for bleeding,**			
Active cancer	2,166 (11%)	11,449 (19%)	0.221
Liver cirrhosis	76 (0.4%)	291 (0.5%)	0.014
Gastroduodenal ulcer	207 (1.1%)	691 (1.2%)	0.008
Anemia	5,393 (28%)	21,316 (36%)	0.166
Leukocyte count >11,000/μL	4,947 (26%)	16,077 (27%)	0.028
Platelet count <100,000/μL	369 (1.9%)	1,626 (2.7%)	0.054
Abnormal prothrombin time	1,437 (8.2%)	4,043 (8.4%)	0.008
CrCl levels <60 mL/min	5,856 (30%)	17,179 (29%)	0.037
**Prognostic scores for bleeding,**			
RIETE, high-risk (≥4 points)	1,630 (9.0%)	5,711 (11%)	0.056
VTE-BLEED, high-risk (≥2 points)	8,859 (47%)	30,055 (52%)	0.098
Modified ACCP, high-risk (≥2 points)	9,497 (53%)	28,325 (52%)	0.009
DOAC, high-risk (≥8 points)	1,418 (10%)	4,133 (10%)	0.006

Abbreviations: SD, standard deviation; BMI, body mass index; DVT, deep vein thrombosis; CrCl, creatinine clearance.
