# Supplementary material for: Low-Density Lipoprotein Cholesterol Levels and Bleeding Risk in Venous Thromboembolism
Source: JAMA Netw Open. 2025 May 9;8(5):e259467. doi: 10.1001/jamanetworkopen.2025.9467 (PMC12065040; doi:10.1001/jamanetworkopen.2025.9467)
Supplement: Supplement 2. — Nonauthor Collaborators. The RIETE Investigators [file jamanetwopen-e259467-s002.pdf]

Supplemental Online Content: Nonauthor Collaborators
*First name, last name, and suffix (if applicable) are required and will appear in PubMed.

***Group Name(s): the RIETE investigators**

*First Name and Middle Initial(s)	*Last Name	*Suffix (eg, Jr, III)	Academic Degrees	Institution	Location (city, state/province, country)	Role or Contribution, eg, chair, principal investigator	Group (if more than 1 Group listed in the byline) and/or Subgroup (eg, Steering Committee)
Manuel	Monreal Bosch		MD, PhD	Faculty of Health Sciences, UCAM - Universidad Católica San Antonio de Murcia. Spain. CIBER Enfermedades Respiratorias (CIBERES)	Badalona, Barcelona, Spain	Chair, Coordinator of the RIETE Registry	Global Coordination of the RIETE Registry
Pedro	Parra Caballero		MD	Department of Internal Medicine, Hospital Universitario de la Princesa	Madrid, Madrid, Spain	Principal Investigator	
Nuria	Ruiz Giménez Arrieta		MD, PhD	Department of Internal Medicine, Hospital Universitario de la Princesa	Madrid, Madrid, Spain	Principal Investigator	
Antonio	López Ruiz		MD	Department of Internal Medicine, Hospital Comarcal de la Axarquía	Vélez-Málaga, Málaga, Spain	Principal Investigator	
Pablo	Demelo Rodríguez		MD, PhD	Department of Internal Medicine, Hospital General Universitario Gregorio Marañón	Madrid, Madrid, Spain	Principal Investigator	
Marta Olimpia	Lago Rodríguez		MD	Department of Internal Medicine, Hospital General Universitario Gregorio Marañón	Madrid, Madrid, Spain	Collaborator	
Marina	López Rubio		MD	Department of Internal Medicine, Hospital General Universitario Gregorio Marañón	Madrid, Madrid, Spain	Collaborator	
Jorge	Del Toro Cervera		MD, PhD	Department of Internal Medicine, Hospital General Universitario Gregorio Marañón	Madrid, Madrid, Spain	Collaborator	
Enric	Grau Segura		MD, PhD	Department of Hematology and Hemotherapy, Hospital Lluis Alcanyís de Xátiva	Xàtiva, Valencia, Spain	Principal Investigator	
Javier	Gutiérrez Guisado		MD, PhD	Department of Internal Medicine, Hospital Monográfico ASEPEYO (de Traumatología Cirugía y Rehabilitación)	Coslada, Madrid, Spain	Principal Investigator	
Luciano	López Jiménez		MD	Department of Internal Medicine, Hospital Universitario Reina Sofía	Córdoba, Córdoba, Spain	Principal Investigator	
María Dolores	Adarraga Cansino		MD, PhD	Department of Internal Medicine, Hospital Universitario Reina Sofía	Córdoba, Córdoba, Spain	Collaborator	
Silvia	Soler Simón		MD	Department of Internal Medicine, Hospital Olot i Comarcal de la Garrotxa	Olot, Gerona, Spain	Principal Investigator	
Alicia	Alda Lozano		MD	Department of Internal Medicine, Hospital Reina Sofía	Tudela, Navarra, Spain	Principal Investigator	
Juan	Criado García		MD, PhD	Department of Internal Medicine, Hospital Universitario Reina Sofía	Córdoba, Córdoba, Spain	Principal Investigator	

Supplemental Online Content: Nonauthor Collaborators
*First name, last name, and suffix (if applicable) are required and will appear in PubMed.

***First Name and Middle Initial(s)**	***Last Name**	***Suffix (eg, Jr, III)**	Academic Degrees	Institution	Location (city, state/province, country)	Role or Contribution, eg, chair, principal investigator	Group (if more than 1 Group listed in the byline) and/or Subgroup (eg, Steering Committee)
Ángeles	Blanco Molina		MD, PhD	Department of Internal Medicine, Hospital Universitario Reina Sofía	Córdoba, Córdoba, Spain	Collaborator	
Ángeles	Fidalgo Fernández		MD, PhD	Department of Internal Medicine, Hospital Universitario de Salamanca	Salamanca, Salamanca, Spain	Principal Investigator	
Remedios	Otero Candelera		MD, PhD	Department of Pneumology, Hospital Universitario Virgen del Rocío	Seville, Seville, Spain	Principal Investigator	
Luis	Jara Palomares		MD	Department of Pneumology, Hospital Universitario Virgen del Rocío	Seville, Seville, Spain	Collaborator	
Iria	Francisco Albesa		MD	Department of Internal Medicine, Hospital Universitari de Girona Dr. Josep Trueta	Gerona, Gerona, Spain	Principal Investigator	
Ana	Alberich Conesa		MD	Department of Internal Medicine, Hospital Universitari de Girona Dr. Josep Trueta	Gerona, Gerona, Spain	Collaborator	
Ferran	García Bragado		MD, PhD	Department of Internal Medicine, Hospital Universitari de Girona Dr. Josep Trueta	Gerona, Gerona, Spain	Collaborator	
Inés	Jou Segovia		MD	Department of Internal Medicine, Hospital Universitari de Girona Dr. Josep Trueta	Gerona, Gerona, Spain	Collaborator	
Carmen	Fernández Capitán		MD, PhD	Department of Internal Medicine, Hospital Universitario La Paz	Madrid, Madrid, Spain	Collaborator	
Giorgina	Salgueiro Origlia		MD	Department of Internal Medicine, Hospital Universitario La Paz	Madrid, Madrid, Spain	Collaborator	
María Isabel	Torres Sánchez		MD	Department of Internal Medicine, Hospital Universitario La Paz	Madrid, Madrid, Spain	Collaborator	
Reina	Valle Bernad		MD, PhD	Department of Internal Medicine, Hospital Sierrallana	Torrelavega, Cantabria, Spain	Principal Investigator	
Cristina	Amado Fernández		MD	Department of Internal Medicine, Hospital Sierrallana	Torrelavega, Cantabria, Spain	Collaborator	
Juan Bosco	López Sáez		MD, PhD	Department of Internal Medicine, Hospital Universitario Puerto Real	Puertoreal, Cádiz, Spain	Principal Investigator	
Montserrat	Pérez Pinar		MD	Department of Internal Medicine, Hospital Virgen de la Luz	Cuenca, Cuenca, Spain	Principal Investigator	
José Antonio	Nieto Rodríguez		MD	Department of Internal Medicine, Hospital Virgen de la Luz	Cuenca, Cuenca, Spain	Collaborator	
Gemma	Vidal Cusidó		MD	Department of Internal Medicine, Hospital de Sabadell	Sabadell, Barcelona, Spain	Principal Investigator	

Supplemental Online Content: Nonauthor Collaborators
*First name, last name, and suffix (if applicable) are required and will appear in PubMed.

*First Name and Middle Initial(s)	*Last Name	*Suffix (eg, Jr, III)	Academic Degrees	Institution	Location (city, state/province, country)	Role or Contribution, eg, chair, principal investigator	Group (if more than 1 Group listed in the byline) and/or Subgroup (eg, Steering Committee)
Laia	Mas Maresma		MD	Department of Internal Medicine, Hospital de Sabadell	Sabadell, Barcelona, Spain	Collaborator	
Fernando	Uresandi Romero		MD, PhD	Deparment of Pneumology, Hospital Universitario Cruces	Barakaldo, Vizcaya, Spain	Collaborator	
Aurora	Villalobos Sánchez		MD, PhD	Department of Internal Medicine, Hospital Universitario Regional de Málaga	Málaga, Málaga, Spain	Principal Investigator	
David	Jiménez Castro		MD, PhD	Department of Pneumology, Hospital Universitario Ramón y Cajal	Madrid, Madrid, Spain	Principal Investigator	
Inmaculada	Cañas Alcántara		MD	Department of Internal Medicine, Hospital General de Granollers	Granollers, Barcelona, Spain	Principal Investigator	
Manuel Jesús	Núñez Fernández		MD	Department of Internal Medicine, Fundación Pública Hospital Comarcal de Salnés	Villagarcia de Arousa, Pontevedra, Spain	Principal Investigator	
Juan Ignacio	Arcelus Martínez		MD, PhD	Department of General Surgery, Hospital Universitario Virgen de las Nieves	Granada, Granada, Spain	Principal Investigator	
Luis Manuel	Hernández Blasco		MD, PhD	Department of Pneumology, Hospital General Universitario Dr. Balmis	Alicante, Alicante, Spain	Principal Investigator	
Ramiro	Cantarella		MD	Department of Pneumology, Hospital General Universitario Dr. Balmis	Alicante, Alicante, Spain	Collaborator	
Patricia	Huélamo López		MD	Department of Pneumology, Hospital General Universitario Dr. Balmis	Alicante, Alicante, Spain	Collaborator	
Manuel	Barrón Medrano		MD	Department of Pneumology, Hospital San Pedro	Logroño, La Rioja, Spain	Principal Investigator	
Belén	Barrón Andrés		N/A	Department of Pneumology, Hospital San Pedro	Logroño, La Rioja, Spain	Collaborator	
Aitor	Ballaz Quincoces		MD	Department of Pneumology, Hospital de Galdakao	Galdakao, Vizcaya, Spain	Principal Investigator	
Javier	Trujillo Santos		MD, PhD	Department of Internal Medicine, Hospital General Universitario Santa Lucía	Cartagena, Murcia, Spain	Principal Investigator	
José María	Pedrajas Navas		MD, PhD	Department of Internal Medicine, Hospital Universitario Clínico San Carlos	Madrid, Madrid, Spain	Principal Investigator	
Vanesa	Sendín Martín		MD	Department of Internal Medicine, Hospital Universitario Clínico San Carlos	Madrid, Madrid, Spain	Collaborator	
José Luis	Fernández Reyes		MD	Department of Internal Medicine, Hospital Universitario de Jaén	Jaén, Jaén, Spain	Principal Investigator	

*First name, last name, and suffix (if applicable) are required and will appear in PubMed.

***First Name and Middle Initial(s)**	***Last Name**	***Suffix (eg, Jr, III)**	Academic Degrees	Institution	Location (city, state/province, country)	Role or Contribution, eg, chair, principal investigator	Group (if more than 1 Group listed in the byline) and/or Subgroup (eg, Steering Committee)
María Isabel	Mercado Montoro		MD	Department of Internal Medicine, Hospital Universitario de Jaén	Jaén, Jaén, Spain	Collaborator	
José	González Martínez		MD	Department of Internal Medicine, Hospital de Sant Joan de Deu (Manresa)	Manresa, Barcelona, Spain	Principal Investigator	
Alicia	Lorenzo Hernández		MD, PhD	Department of Internal Medicine, Hospital Universitario La Paz	Madrid, Madrid, Spain	Principal Investigator	
Teresa	Sancho Bueso		MD	Department of Internal Medicine, Hospital Universitario La Paz	Madrid, Madrid, Spain	Collaborator	
Ramón	Puchades Rincón de Arellano		MD, PhD	Department of Internal Medicine, Hospital Universitario La Paz	Madrid, Madrid, Spain	Collaborator	
José Antonio	Porras Ledantes		MD, PhD	Department of Internal Medicine, Hospital Univ. Joan XXIII de Tarragona	Tarragona, Tarragona, Spain	Principal Investigator	
Pablo Javier	Marchena Yglesias		MD	Department of Internal Medicine, Parc Sanitari Sant Joan de Deu - Hospital General	Sant Boi de Llobregat, Barcelona, Spain	Principal Investigator	
Leticia	Guirado Torrecilla		MD, PhD	Department of Internal Medicine, Hospital Clínico Universitario Virgen de la Arrixaca	El Palmar, Murcia, Spain	Principal Investigator	
Gabriel	Puche Palao		MD	Department of Internal Medicine, Hospital Clínico Universitario Virgen de la Arrixaca	El Palmar, Murcia, Spain	Collaborator	
Sonia	Otálora Valderrama		MD, PhD	Department of Internal Medicine, Hospital Clínico Universitario Virgen de la Arrixaca	El Palmar, Murcia, Spain	Collaborator	
Olga	Madridano Cobo		MD, PhD	Department of Internal Medicine, Hospital Universitario Infanta Sofía	San Sebastián de los Reyes, Madrid, Spain	Principal Investigator	
Jorge Manuel	Maza Ortiz		MD	Department of Internal Medicine, Hospital Universitario Infanta Sofía	San Sebastián de los Reyes, Madrid, Spain	Collaborator	
Mar	Martín del Pozo		MD, PhD	Department of Internal Medicine, Hospital Universitario Infanta Sofía	San Sebastián de los Reyes, Madrid, Spain	Principal Investigator	
Irene	Cabello Rojano		MD	Department of Internal Medicine, Hospital Universitario Infanta Sofía	San Sebastián de los Reyes, Madrid, Spain	Collaborator	
Iciar Agurtzane	Ibáñez Sustacha		MD	Department of Internal Medicine, Hospital Universitario Infanta Sofía	San Sebastián de los Reyes, Madrid, Spain	Collaborator	
María del Valle	Morales Gavilán		MD	Department of Internal Medicine, Hospital del Tajo	Aranjuez, Madrid, Spain	Principal Investigator	

Supplemental Online Content: Nonauthor Collaborators
*First name, last name, and suffix (if applicable) are required and will appear in PubMed.

*First Name and Middle Initial(s)	*Last Name	*Suffix (eg, Jr, III)	Academic Degrees	Institution	Location (city, state/province, country)	Role or Contribution, eg, chair, principal investigator	Group (if more than 1 Group listed in the byline) and/or Subgroup (eg, Steering Committee)
Ana	Maestre Peiró		MD, PhD	Deparment of Internal Medicine, Hospital Universitario de Vinalopó	Elche, Alicante, Spain	Principal Investigator	
José Carlos	Escribano Stable		MD	Deparment of Internal Medicine, Hospital Universitario de Vinalopó	Elche, Alicante, Spain	Collaborator	
Mª Luisa	Peris Sifre		MD, PhD	Department of Internal Medicine, Consorcio Hospitalario Provincial de Castellón	Castellón de la Plana, Castellón, Spain	Principal Investigator	
Raquel	Barba Martín		MD, PhD	Deparment of Internal Medicine, Hospital Universitario Rey Juan Carlos	Móstoles, Madrid, Spain	RIETE National Coordinator (Spain), Principal Investigator	RIETE National Coordination
María Dolores	Joya Seijo		MD	Deparment of Internal Medicine, Hospital Universitario Rey Juan Carlos	Móstoles, Madrid, Spain	Collaborator	
María	Angelina García		MD, PhD	Deparment of Internal Medicine, Hospital Universitario Rey Juan Carlos	Móstoles, Madrid, Spain	Collaborator	
Aída	Gil Díaz		MD	Department of Internal Medicine, Hospital Universitario de Gran Canaria Dr. Negrín	Las Palmas de Gran Canaria, Las Palmas, Spain	Principal Investigator	
Paula	Villares Fernández		MD, PhD	Department of Internal Medicine, Hospital Universitario HM Sanchinarro	Madrid, Madrid, Spain	Principal Investigator	
Laura	Monzón Escribano		MD	Department of Internal Medicine, Hospital Universitario HM Sanchinarro	Madrid, Madrid, Spain	Collaborator	
Ana	Rodríguez Cobo		MD, PhD	Department of Internal Medicine, Hospital Universitario HM Sanchinarro	Madrid, Madrid, Spain	Collaborator	
José Antonio	Díaz Peromingo		MD	Department of Internal Medicine, Hospital Clínico Universitario de Santiago	Santiago de Compostela, A Coruña, Spain	Principal Investigator	
Alberto	García Ortega		MD, PhD	Department of Pneumology, Hospital Universitario Doctor Peset	Valencia, Valencia, Spain	Principal Investigator	
Ana	Pedro Tudela		MD	Department of Pneumology, Hospital Universitario Doctor Peset	Valencia, Valencia, Spain	Collaborator	
Laura	Taberner Lino		MD	Department of Pneumology, Hospital Universitario Doctor Peset	Valencia, Valencia, Spain	Collaborator	
Olga	Gavín Sebastián		MD, PhD	Department of Hematology, Hospital Clínico Universitario Lozano Blesa	Zaragoza, Zaragoza, Spain	Principal Investigator	
Covadonga	Gómez Cuervo		MD	Department of Internal Medicine, Hospital Universitario 12 de Octubre	Madrid, Madrid, Spain	Principal Investigator	

Supplemental Online Content: Nonauthor Collaborators
*First name, last name, and suffix (if applicable) are required and will appear in PubMed.

*First Name and Middle Initial(s)	*Last Name	*Suffix (eg, Jr, III)	Academic Degrees	Institution	Location (city, state/province, country)	Role or Contribution, eg, chair, principal investigator	Group (if more than 1 Group listed in the byline) and/or Subgroup (eg, Steering Committee)
Jesús	González Olmedo		MD	Department of Internal Medicine, Hospital Universitario 12 de Octubre	Madrid, Madrid, Spain	Collaborator	
Pilar	Parra Rosado		MD	Department of Internal Medicine, Hospital Universitario 12 de Octubre	Madrid, Madrid, Spain	Collaborator	
Pilar	Llamas Sillero		MD	Department of Hematology, Hospital Universitario Fundación Jiménez Díaz	Madrid , Madrid, Spain	Principal Investigator	
Gonzalo	Castellanos Arias		MD	Department of Hematology, Hospital Universitario Fundación Jiménez Díaz	Madrid , Madrid, Spain	Collaborator	
Juan Carlos	Caballero Hernáez		MD	Department of Hematology, Hospital Universitario Fundación Jiménez Díaz	Madrid , Madrid, Spain	Collaborator	
María Lourdes	Pesce		MD	Department of Internal Medicine, Hospital General Universitario de Elda	Elda, Alicante , Spain	Principal Investigator	
Patricia	López Miguel		MD	Department of Pneumology, Hospital General Universitario de Albacete	Albacete, Albacete, Spain	Principal Investigator	
Juan José	López Núñez		MD, PhD	Department of Internal Medicine, Hospital Germans Trias i Pujol	Badalona , Barcelona, Spain	Principal Investigator	
Jesús	Aibar Gallizo		MD, PhD	Department of Internal Medicine, Hospital Universitario Clínic de Barcelona	Barcelona, Barcelona, Spain	Principal Investigator	
María	Ortiz Rodríguez		MD	Department of Internal Medicine, Hospital Universitario Clínic de Barcelona	Barcelona, Barcelona, Spain	Collaborator	
Cristina	Gabara Xanco		MD	Department of Internal Medicine, Hospital Universitario Clínic de Barcelona	Barcelona, Barcelona, Spain	Collaborator	
Cristina	Barbagelata López		MD	Department of Internal Medicine, Complexo Hospitalario Universitario A Coruña	A Coruña, A Coruña, Spain	Principal Investigator	
Álvaro	Dubois Silva		MD	Department of Internal Medicine, Complexo Hospitalario Universitario A Coruña	A Coruña, A Coruña, Spain	Collaborator	
Joaquin	Alfonso Megido		MD	Department of Internal Medicine, Hospital Valle del Nalón	Langreo, Asturias, Spain	Principal Investigator	
Susana	Suárez Fernández		MD	Department of Intensive Medicine, Hospital Valle del Nalón	Langreo, Asturias, Spain	Collaborator	
María del Carmen	Díaz Pedroche		MD, PhD	Department of Internal Medicine, Hospital Universitario 12 de Octubre	Madrid, Madrid, Spain	Principal Investigator	

Supplemental Online Content: Nonauthor Collaborators
*First name, last name, and suffix (if applicable) are required and will appear in PubMed.

***First Name and Middle Initial(s)**	***Last Name**	***Suffix (eg, Jr, III)**	Academic Degrees	Institution	Location (city, state/province, country)	Role or Contribution, eg, chair, principal investigator	Group (if more than 1 Group listed in the byline) and/or Subgroup (eg, Steering Committee)
José	Bascuñana Morejón de Girón		MD	Department of Internal Medicine, Hospital Universitario 12 de Octubre	Madrid, Madrid, Spain	Collaborator	
Paloma	Agudo de Blas		MD, PhD	Department of Internal Medicine, Hospital Universitario 12 de Octubre	Madrid, Madrid, Spain	Collaborator	
Francisco	Rivera Cívico		MD	Department of Internal Medicine, Hospital de Poniente	El Ejido, Almeria, Spain	Principal Investigator	
Ana María	Díaz Brasero		MD	Department of Internal Medicine, Hospital Universitario de Guadalajara	Guadalajara, Guadalajara, Spain	Principal Investigator	
Sara	Láinez Justo		MD	Department of Internal Medicine, Hospital Universitario de Guadalajara	Guadalajara, Guadalajara, Spain	Collaborator	
Carolina	Gómez Cepeda		MD	Department of Internal Medicine, Hospital Universitario de Guadalajara	Guadalajara, Guadalajara, Spain	Collaborator	
Alberto	Rivera Gallego		MD, PhD	Department of Internal Medicine, Complexo Hospitalario Universitario de Vigo	Vigo, Pontevedra, Spain	Principal Investigator	
José Felipe	Varona Arche		MD, PhD	Department of Internal Medicine, Hospital Universitario HM Montepríncipe	Boadilla del Monte, Madrid, Spain	Principal Investigator	
Nazaret	Pacheco Gómez		MD	Department of Internal Medicine, Hospital San Pedro de Alcántara	Cáceres, Cáceres, Spain	Principal Investigator	
Gisela	Claver Cercós		MD	Department of Internal Medicine, Hospital Sant Camil	Sant Pere de Ribes, Barcelona, Spain	Principal Investigator	
Agustina	Rivas Guerrero		MD	Department of Pneumology, Hospital Universitario de Donostia	San Sebastián, Guipúzcoa, Spain	Principal Investigator	
Javier	Pagán Escribano		MD, PhD	Department of Internal Medicine, Hospital General Universitario Morales Meseguer	Murcia, Murcia, Spain	Principal Investigator	
David	Vicente Navarro		MD	Department of Internal Medicine, Hospital Universitario de Torrevieja	Torrevieja, Alicante , Spain	Principal Investigator	
Adriana	González Munera		MD, PhD	Department of Internal Medicine, Hospital Universitario Vithas Madrid La Milagrosa	Madrid, Madrid, Spain	Principal Investigator	
Juan	Gorostidi Pérez		MD	Department of Pneumology, Hospital Universitario de Cabueñes	Gijón, Asturias, Spain	Principal Investigator	
Ana Rosa	Expósito Villegas		MD	Department of Pneumology, Hospital Universitario de Cabueñes	Gijón, Asturias, Spain	Collaborator	

*First name, last name, and suffix (if applicable) are required and will appear in PubMed.

***First Name and Middle Initial(s)**	***Last Name**	***Suffix (eg, Jr, III)**	Academic Degrees	Institution	Location (city, state/province, country)	Role or Contribution, eg, chair, principal investigator	Group (if more than 1 Group listed in the byline) and/or Subgroup (eg, Steering Committee)
Irene	Gorostidi Álvarez		MD	Department of Pneumology, Hospital Universitario de Cabueñes	Gijón, Asturias, Spain	Collaborator	
Diego	Durán Barata		MD	Department of Pneumology, Hospital Universitario de Getafe	Getafe, Madrid, Spain	Principal Investigator	
María del Carmen	Olivares Hidalgo		MD	Department of Internal Medicine, Hospital de Tortosa Verge de la Cinta	Tortosa, Tarragona, Spain	Principal Investigator	
Clàudia Salomé	Bonavila Juan		MD	Department of Internal Medicine, Hospital de Tortosa Verge de la Cinta	Tortosa, Tarragona, Spain	Collaborator	
Emma	Vázquez Espinosa		MD, PhD	Department of Pneumology, Hospital Universitario de la Princesa	Madrid, Madrid, Spain	Principal Investigator	
Cihan	Ay		MD	Department of Hematology and Haemostaseology, Medical University of Vienna	Vienna, Vienna, Austria	Principal Investigator	
Stephan	Nopp		MD, PhD	Department of Hematology and Haemostaseology, Medical University of Vienna	Vienna, Vienna, Austria	Collaborator	
Ingrid	Pabinger		MD	Department of Hematology and Haemostaseology, Medical University of Vienna	Vienna, Vienna, Austria	Collaborator	
Peter	Verhamme		MD, PhD	Department of Vascular Medicine and Haemostasis, University of Leuven	Leuven, Leuven, Belgium	RIETE National Coordinator (Belgium), Principal Investigator	RIETE National Coordination
Quentin	Van Thillo		MD, PhD	Department of Vascular Medicine and Haemostasis, University of Leuven	Leuven, Leuven, Belgium	Collaborator	
Andreas	Verstraete		MD	Department of Vascular Medicine and Haemostasis, University of Leuven	Leuven, Leuven, Belgium	Collaborator	
Hugo Hyung Bok	Yoo		MD	Department of Internal Medicine - Pulmonary Division, Botucatu Medical School - São Paulo State University (UNESP)	Botucatu, São Paulo, Brazil	RIETE National Coordinator (Brazil), Principal Investigator	RIETE National Coordination
Ana Cristina	Montenegro Arenas		MD	Department of Vascular Medicine, Hospital Universitario Fundacion Santa Fe de Bogota	Bogotá, Cundinamarca, Colombia	Principal Investigator	

*First name, last name, and suffix (if applicable) are required and will appear in PubMed.

***First Name and Middle Initial(s)**	***Last Name**	***Suffix (eg, Jr, III)**	Academic Degrees	Institution	Location (city, state/province, country)	Role or Contribution, eg, chair, principal investigator	Group (if more than 1 Group listed in the byline) and/or Subgroup (eg, Steering Committee)
Carlos Andrés	Jiménez Echandía		MD	Department of Vascular Medicine, Hospital Universitario Fundacion Santa Fe de Bogota	Bogotá, Cundinamarca, Colombia	Collaborator	
Jairo	Roa		MD	Department of Internal Medicine, Hospital Universitario Fundacion Santa Fe de Bogota	Bogotá, Cundinamarca, Colombia	Collaborator	
Radovan	Malý		MD, PhD	Department of Cardiovascular Medicine I, University Hospital Hradec Kralove	Hradec Králové, Czech Republic	RIETE National Coordinator (Czech Republic), Principal Investigator	RIETE National Coordination
Jana	Hirmerova		MD, PhD	Department of Internal Medicine, University Hospital Plzen	Plzen - Bory, Plzen, Czech Republic	Principal Investigator	
Géraldine	Poenou		MD	Department of Medicine and Therapeutics, Hôpital Nord - CHU St-Etienne	Saint-Etienne, Saint-Etienne, France	Principal Investigator	
Sandrine	Accassat		MD	Department of Medicine and Therapeutics, Hôpital Nord - CHU St-Etienne	Saint-Etienne, Saint-Etienne, France	Collaborator	
Laurent	Bertoletti		MD, PhD	Department of Vascular Medicine and Therapeutics, Hôpital Nord - CHU St-Etienne	Saint-Etienne, Saint-Etienne, France	RIETE National Coordinator (France), Principal Investigator	RIETE National Coordination
Alessandra	Bura-Riviere		MD, PhD	Department of Vascular Medicine, Hôpital de Rangueil	Toulouse, Toulouse, France	Principal Investigator	
Isabelle	Quéré		MD, PhD	Department of Vascular Medicine, Hôpital Saint Eloi	Montpellier, Montpellier, France	Principal Investigator	
Isabelle	Mahé		MD, PhD	Department of Internal Medicine, Hôpital Louis Mourier	Colombes, Colombes, France	Principal Investigator	
Ludovic	Plaisance		MD	Department of Internal Medicine, Hôpital Louis Mourier	Colombes, Colombes, France	Collaborator	
Edouard	Versini		MD	Department of Internal Medicine, Hôpital Louis Mourier	Colombes, Colombes, France	Collaborator	
Farès	Moustafa		MD	Department of Emergency, Clermont-Ferrand University Hospital	Clermont-Ferrand, Clermont-Ferrand, France	Principal Investigator	
Gabrielle	Sarlon-Bartoli		MD, PhD	Department of Vascular Medicine and Arterial Hypertension, Hôpital de la Timone	Marseille, Marseille, France	Principal Investigator	

Supplemental Online Content: Nonauthor Collaborators
*First name, last name, and suffix (if applicable) are required and will appear in PubMed.

***First Name and Middle Initial(s)**	***Last Name**	***Suffix (eg, Jr, III)**	Academic Degrees	Institution	Location (city, state/province, country)	Role or Contribution, eg, chair, principal investigator	Group (if more than 1 Group listed in the byline) and/or Subgroup (eg, Steering Committee)
Barbara	Leclercq		MD	Department of Vascular Medicine and Arterial Hypertension, Hôpital de la Timone	Marseille, Marseille, France	Collaborator	
Pierre	Suchon		MD, PhD	Department of Vascular Medicine and Arterial Hypertension, Hôpital de la Timone	Marseille, Marseille, France	Collaborator	
Olivier	Espitia		MD, PhD	Department of Internal Medicine, CHU Nantes	Nantes, Nantes, France	Principal Investigator	
Judith	Catella		MD	Department of Internal Medicine, Hôpital Édouard Herriot	Lyon, Lyon, France	Principal Investigator	
Romain	Chopard		MD, PhD	Department of Cardiology, University Hospital Jean Minjoz	Besançon, Besançon, France	Principal Investigator	
Sebastian	Schellong		MD, PhD	Department of Medical Clinic II, Municipal Hospital of Dresden Friedrichstadt	Friedrichstadt, Dresden, Germany	RIETE National Coordinator (Germany), Principal Investigator	RIETE National Coordination
Benjamin	Brenner		MD	Department of Hematology, Rambam Health Care Campus	Haifa, Haifa, Israel	RIETE Steering Committee Member, Principal Investigator	RIETE Steering Committee
Inna	Tzoran		MD, PhD	Department of Hematology, Rambam Health Care Campus	Haifa, Haifa, Israel	RIETE National Coordinator (Israel), Principal Investigator	RIETE National Coordination
Gili	Kenet		MD	Department of Pediatric Hematology-Oncology, Sheba Medical Center	Ramat Gan, Tel Hashomer, Israel	Principal Investigator	
Parham	Sadeghipour		MD	Department of Peripheral Vascular Diseases, Rajaie Cardiovascular Medical and Research Center	Tehran, Tehran, Iran	Principal Investigator	
Bahram	Mohebbi		MD, PhD	Department of Peripheral Vascular Diseases, Rajaie Cardiovascular Medical and Research Center	Tehran, Tehran, Iran	Collaborator	
Yaser	Jenab		MD	Department of Cardiology, Tehran Heart Center	Tehran, Tehran, Iran	Principal Investigator	
Alireza	Khodayari Jvazma		MD	Department of Cardiology, Tehran Heart Center	Tehran, Tehran, Iran	Collaborator	

Supplemental Online Content: Nonauthor Collaborators
*First name, last name, and suffix (if applicable) are required and will appear in PubMed.

***First Name and Middle Initial(s)**	***Last Name**	***Suffix (eg, Jr, III)**	Academic Degrees	Institution	Location (city, state/province, country)	Role or Contribution, eg, chair, principal investigator	Group (if more than 1 Group listed in the byline) and/or Subgroup (eg, Steering Committee)
Somayeh	Yadangi		BSN	Department of Cardiology, Tehran Heart Center	Tehran, Tehran, Iran	Collaborator	
Farid	Rashidi		MD	Department of Pulmonary and Critical Care, Imam Reza Hospital	Tabriz, Azerbaiyán Oriental, Iran	Principal Investigator	
Pierpaolo	Di Micco		MD, PhD	Department of Internal Medicine and Emergency Room, Ospedale Buon Consiglio Fatebenefratelli	Naples, Naples, Italy	RIETE National Coordinator (Italy), Principal Investigator	RIETE National Coordination
Paolo	Prandoni		MD, PhD	Department of Cardiovascular Sciences. Vascular Medicine Unit, University of Padua	Padua, Padua, Italy	RIETE Steering Committee Member, Principal Investigator	RIETE Steering Committee
Franca	Bilora		MD, PhD	Department of Cardiovascular Sciences. Vascular Medicine Unit, University of Padua	Padua, Padua, Italy	Collaborator	
Paolo	Simioni		MD, PhD	Department of Cardiovascular Sciences. Vascular Medicine Unit, University of Padua	Padua, Padua, Italy	Collaborator	
Carmine	Siniscalchi		MD, PhD	Department of Angiology, Azienda Ospedaliera Universitaria di Parma	Parma, Parma, Italy	Principal Investigator	
Manuela	Basaglia		MD	Department of Angiology, Azienda Ospedaliera Universitaria di Parma	Parma, Parma, Italy	Collaborator	
Barbara	Brandolin		MD	Department of Vascular Medicine, Ospedale CastelFranco Veneto	Castelfranco, Castelfranco Veneto, Italy	Collaborator	
Beniamino	Zalunardo		MD	Department of Vascular Medicine, Ospedale CastelFranco Veneto	Castelfranco, Castelfranco Veneto, Italy	Collaborator	
Tatiana	Scandiuzzi Piovesan		MD	Department of Clinical Medicine, Ospedale CastelFranco Veneto	Castelfranco, Castelfranco Veneto, Italy	Collaborator	
Giovanni	Barillari		MD, PhD	Department of Hemorrhagic and Thrombotic Diseases, General and University Hospital S. Maria della Misericordia	Udine, Udine, Italy	Principal Investigator	
Alessandra	Poz		MD, PhD	Department of Hemorrhagic and Thrombotic Diseases, General and University Hospital S. Maria della Misericordia	Udine, Udine, Italy	Collaborator	

Supplemental Online Content: Nonauthor Collaborators
*First name, last name, and suffix (if applicable) are required and will appear in PubMed.

*First Name and Middle Initial(s)	*Last Name	*Suffix (eg, Jr, III)	Academic Degrees	Institution	Location (city, state/province, country)	Role or Contribution, eg, chair, principal investigator	Group (if more than 1 Group listed in the byline) and/or Subgroup (eg, Steering Committee)
Maurizio M	Ciammaichella		MD	Department of Emergency Internal Medicine, Ospedale St. John	Roma, Roma, Italy	Principal Investigator	
Beldisa	Taflaj		MD	Department of Emergency Internal Medicine, Ospedale St. John	Roma, Roma, Italy	Collaborator	
Cristiano	Bortoluzzi		MD	Department of Internal Medicine - Unit of Angiology, Ospedale SS. Giovanni e Paolo di Venezia	Venice, Venice, Italy	Principal Investigator	
Matteo	Bortoluzzi		MD	Department of Internal Medicine - Unit of Angiology, Ospedale SS. Giovanni e Paolo di Venezia	Venice, Venice, Italy	Collaborator	
Paolo	Scarinzi		MD	Department of Internal Medicine - Unit of Angiology, Ospedale SS. Giovanni e Paolo di Venezia	Venice, Venice, Italy	Collaborator	
Egidio	Imbalzano		MD	Department of Clinical and Experimental Medicine, A.O.U Policlinico “G. Martino”	Messina, Messina, Italy	Principal Investigator	
Matteo	Giorgi-Pierfranceschi		MD	Department of Internal Medicine, Istituti Ospitalieri di Cremona	Cremona, Cremona, Italy	Principal Investigator	
Abramo	Bazza		MD	Department of Internal Medicine, Istituti Ospitalieri di Cremona	Cremona, Cremona, Italy	Collaborator	
Daniele	Bissacco		MD	Department of Vascular Surgery, IRCCS Istituto Auxologico Italiano	Milan, Milan, Italy	Principal Investigator	
Renato	Casana		MD	Department of Vascular Surgery, IRCCS Istituto Auxologico Italiano	Milan, Milan, Italy	Collaborator	
Daniela	Lambertenghi-Deliliers		MD	Department of Vascular Surgery, IRCCS Istituto Auxologico Italiano	Milan, Milan, Italy	Collaborator	
Andris	Skride		MD, PhD	Department of Cardiology, Pauls Stradins Clinical University Hospital	Riga, Riga, Latvia	Principal Investigator	
Daniela Dana	Bizika		MD	Department of Cardiology, Pauls Stradins Clinical University Hospital	Riga, Riga, Latvia	Collaborator	
Rolands	Paluga		MD	Department of Cardiology, Pauls Stradins Clinical University Hospital	Riga, Riga, Latvia	Collaborator	
Zoubida	Tazi Mezalek		MD	Department of Internal Medicine, Ibn Sina Hospital	Rabat, Rabat, Morocco	Principal Investigator	

Supplemental Online Content: Nonauthor Collaborators
*First name, last name, and suffix (if applicable) are required and will appear in PubMed.

***First Name and Middle Initial(s)**	***Last Name**	***Suffix (eg, Jr, III)**	Academic Degrees	Institution	Location (city, state/province, country)	Role or Contribution, eg, chair, principal investigator	Group (if more than 1 Group listed in the byline) and/or Subgroup (eg, Steering Committee)
Marijan	Bosevski		MD, PhD	University Cardiology Clinic, Faculty of Medicine	Skopje, Skopje, North Macedonia	RIETE National Coordinator (R. North Macedonia), Principal Investigator	RIETE National Coordination
Marija	Trajkova		MD	University Cardiology Clinic, Faculty of Medicine	Skopje, Skopje, North Macedonia	Collaborator	
Jose	Meireles		MD	Department of Internal Medicine, Unidade Local de Saúde Entre Douro e Vouga	Santa María da Feira, Santa María da Feira, Portugal	Principal Investigator	
Rafael	Marques		MD	Department of Internal Medicine, Unidade Local de Saúde Entre Douro e Vouga	Santa María da Feira, Santa María da Feira, Portugal	Collaborator	
Samuel	Fonseca		MD	Department of Internal Medicine, Unidade Local de Saúde Entre Douro e Vouga	Santa María da Feira, Santa María da Feira, Portugal	Collaborator	
Lucia	Mazzolai		MD, PhD	Department of Angiology, Centre Hospitalier Universitaire Vaudois (CHUV)	Lausanne, Lausanne, Switzerland	RIETE National Coordinator (Switzerland), Principal Investigator	RIETE National Coordination
Stefano	Barco		MD, PhD	Department of Angiology, University Hospital Zurich	Zurich, Zurich, Switzerland	Principal Investigator	
Avinash	Aujayeb		MBBS	Department of Respiratory Medicine, Northumbria Healthcare NHS Foundation Trust	Newcastle upon Tyne, Tyne y Wear, United Kingdom	Principal Investigator	
Alfonso J	Tafur		MD, MS	Department of Medicine and Vascular Medicine, Evanston NorthShore University HealthSystem	Evanston, Illinois, Chicago, USA	Principal Investigator	
Joseph A	Caprini		MD	Department of Medicine and Vascular Medicine, Evanston NorthShore University HealthSystem	Evanston, Illinois, Chicago, USA	RIETE National Coordinator (USA), Collaborator	RIETE National Coordination
Ido	Weinberg		MD	Department of Cardiology, Massachusetts General Hospital	Boston, Massachusetts , USA	Principal Investigator	
Abdurahman	Khalil		MD	Department of Cardiology, Massachusetts General Hospital	Boston, Massachusetts , USA	Collaborator	

Supplemental Online Content: Nonauthor Collaborators
*First name, last name, and suffix (if applicable) are required and will appear in PubMed.

***First Name and Middle Initial(s)**	***Last Name**	***Suffix (eg, Jr, III)**	Academic Degrees	Institution	Location (city, state/province, country)	Role or Contribution, eg, chair, principal investigator	Group (if more than 1 Group listed in the byline) and/or Subgroup (eg, Steering Committee)
Luis	Ortega Paz		MD, PhD	Department of Medicine - Division of Cardiology, College of Medicine – Jacksonville; University of Florida	Jacksonville, Florida, USA	Principal Investigator	
Dominick J	Angiolillo		MD, PhD	Department of Medicine - Division of Cardiology, College of Medicine – Jacksonville; University of Florida	Jacksonville, Florida, USA	Collaborator	
Hanh My	Bui		MD, PhD	Department of Scientific Research Management, Hanoi Medical University Hospital	Hanoi, Hanoi, Vietnam	RIETE National Coordinator (Vietnam), Principal Investigator	RIETE National Coordination
Dominique	Farge-Bancel		MD, PhD	Department of Internal Medicine and Pathology, Hôpital Saint-Louis	Paris, Paris, France	RIETE Steering Committee Member	RIETE Steering Committee
Abílio	Reis		MD, PhD	Department of Medicine, Coordinator of Pulmonary Vascular Diseases Unit, Centro Hospitalar Universitário do Porto	Porto, Portugal	RIETE National Coordinator (Portugal)	RIETE National Coordination
