# Supplementary material for: Low-Density Lipoprotein Cholesterol Levels and Bleeding Risk in Venous Thromboembolism
Source: JAMA Netw Open. 2025 May 9;8(5):e259467. doi: 10.1001/jamanetworkopen.2025.9467 (PMC12065040; doi:10.1001/jamanetworkopen.2025.9467)
Supplement: Supplement 3. — Data Sharing Statement [file jamanetwopen-e259467-s003.pdf]

Data Sharing Statement

Siniscalchi. Low-Density Lipoprotein Cholesterol Levels and Bleeding Risk in Venous Thromboembolism. *JAMA Netw Open*. Published May 09, 2025.
doi:10.1001/jamanetworkopen.2025.9467

Data
Data available: No
